# Why and how to open intensive care units to family visits during the pandemic

**DOI:** 10.1186/s13054-021-03608-3

**Published:** 2021-06-02

**Authors:** Giovanni Mistraletti, Alberto Giannini, Giuseppe Gristina, Paolo Malacarne, Davide Mazzon, Elisabetta Cerutti, Alessandro Galazzi, Ilaria Giubbilo, Marco Vergano, Vladimiro Zagrebelsky, Luigi Riccioni, Giacomo Grasselli, Silvia Scelsi, Maurizio Cecconi, Flavia Petrini

**Affiliations:** 1 Department of Pathophysiology and Transplantation, Università degli Studi di Milano, Milan, Italy; 2 SC Anesthesia and Intensive Care, San Paolo Hospital - Polo Universitario, ASST Santi Paolo e Carlo, Milan, Italy; 3 Unit of Pediatric Anesthesia and Intensive Care, Children’s Hospital, ASST Spedali Civili, Brescia, Italy; 4 Italian Society of Anaesthesia, Analgesia, Reanimation, and Intensive Care Medicine (SIAARTI) Ethics, Rome, Italy; 5 U.O. Anesthesia and Intensive Care, AOU Pisana, Pisa, Italy; 6 UOC Anesthesia and Intensive Care, Belluno Hospital, Belluno, Italy; 7 Department of Anesthesia and Transplant, Surgical Intensive Care, AOU Ospedali Riuniti, Ancona, Italy; 8 Direction of Healthcare Professions, Fondazione IRCCS Ca’ Granda Ospedale Maggiore Policlinico, Milan, Italy; 9 General and Neurosurgical ICU, Ospedale dell’Angelo, AULSS 3 Serenissima Veneto, Venice, Italy; 10 Department of Anesthesia and Intensive Care, San Giovanni Bosco Hospital, Turin, Italy; 11 Director, Laboratorio dei Diritti Fondamentali, Collegio Carlo Alberto, Turin, Italy; 12 ICU 4, AO San Camillo-Forlanini, Rome, Italy; 13 Department of Anesthesia, Critical Care and Emergency, Fondazione IRCCS Ca’ Granda Ospedale Maggiore Policlinico, Milan, Italy; 14 Chair Aniarti, Director of Health Profession Department, IRCCS Istituto Giannina Gaslini, Genoa, Italy; 15 Department of Biomedical Sciences, Humanitas University, Pieve Emanuele, Italy; 16 Department of Anesthesia and Intensive Care, IRCCS Humanitas Research Hospital, Rozzano, Italy; 17 SIAARTI President - Retired Full Professor of Anesthesia and Intensive Care, Chieti-Pescara University, Chieti, Italy

**Keywords:** Professional/family relations, Social isolation, Health communication, Information dissemination, Family health, Intensive care units, Communicable diseases, Pandemics

## Abstract

Since the lockdown because of the pandemic, family members have been prohibited from visiting their loved ones in hospital. While it is clearly complicated to implement protocols for the admission of family members, we believe precise strategic goals are essential and operational guidance is needed on how to achieve them. Even during the pandemic, we consider it a priority to share strategies adapted to every local setting to allow family members to enter intensive care units and all the other hospital wards.

**Supplementary Information:**

The online version contains supplementary material available at 10.1186/s13054-021-03608-3.

## Background

The pandemic of severe acute respiratory syndrome corona virus 2 (SARS-CoV-2), abruptly interrupted a decades-long path of "humanization" [1, 2] and "opening" of intensive care units (ICUs) [3, 4]. This is a precise need and clearly a right of patients and their families [5], establishing understanding and collaboration between families and the care team.

At the beginning of the pandemic, regulations were issued prohibiting family members from being physically close to their loved ones in hospital, mainly because of the scarcity of personal protective equipment (PPE) and limited knowledge about the disease and its transmission. These rules, which are still in force or are only slightly less restrictive a year later, are often perceived as unjust by people who have an understandable desire to be close to their loved ones, especially during the end-of-life stages [6, 7].

Many current regional provisions strictly restrict family visits. However, entry into hospitals is allowed in situations of “particular frailty and vulnerability of hospitalized patients” [8] or in any case “of special need” [9, 10], with strict limits on the time and number of visitors. The same conditions apply to pediatric wards and pediatric ICUs. It is left to the ward head to decide in which cases exceptions are appropriate.

The situation in corona virus disease (CoViD) wards remains extremely challenging. While we are aware of the complexity involved in implementing protocols for the admission of family members, we believe it is absolutely essential to set some precise strategic goals [11] and provide operational guidance on how to achieve them. Even during the pandemic, knowing that we cannot guarantee immediate results, we nevertheless consider it a priority to find shared strategies adaptable to every local setting to allow family members to enter CoViD wards [12].

The aim of the present paper is to share our viewpoint and experience about the benefits of permitting visits in ICUs during a pandemic period, to identify barriers to the relatives’ presence, and to discuss possible strategies to overcome them. The reasons leading our beliefs are presented in the Electronic Supplementary Material, together with a presentation of the Authors characteristics [Additional file 1].

## Benefits for patients, relatives, and healthcare team

The therapeutic choices that guide the care of each patient, to be effective, respectful and proportionate, call for sharing between the patient, family members and health professionals [13], even in times of pandemic [14]. First and foremost, this implies appropriate communication by telephone or video calls [15], which are also possible towards the end of life [16]. These communication modalities, even if feasible, are not sufficient [17] and pose some objective difficulties, including respect for privacy and confidentiality [18]. The physical presence of family members makes it simpler to share care pathways: It permits more effective information [19], greater transparency and better understanding of decision-making processes, and makes sharing care choices more feasible.

Clinically speaking, the presence of family members offers relational benefits particularly at the end of the deep sedation phase and during prolonged non-invasive mechanical ventilation: it can significantly help reduce the prevalence of delirium [20], which is higher in CoViD patients than in other critically ill patients [21].

The presence of family members strongly motivates the patient to continue necessary but burdensome care. Even if limited in time and conditioned by the necessary PPE, it responds to a patient’s need, boosts the family members’ trust and appreciation of the care team [22], limits the understandable difficulty for family members to accept bad news, without actually having been able to verify directly what it implies in organizational and care terms to treat and care for critical patients.

The physical presence of family members helps protect them against “complicated grief.” Rarely in recent history conditions have arisen where it is impossible to be close to loved ones at their death. This custom is extremely ingrained worldwide [7]. The possibility of being physically close to one’s loved one even at the moment of death [23], if requested by family members, helps reduce the risk of psychological issues developing [24], which can persist for a long time.

**Fig. 1:**
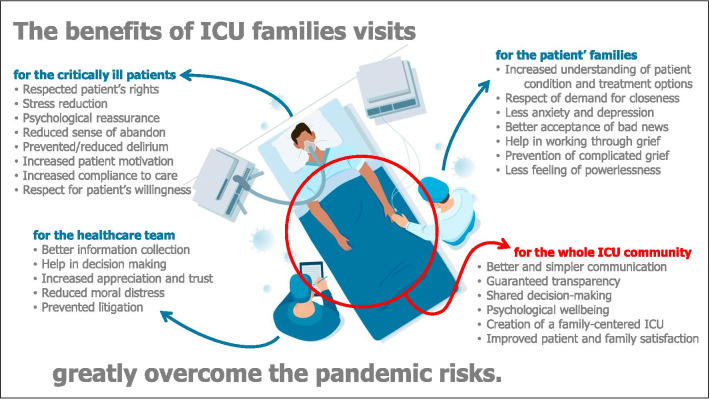
The physical presence of families into the ICU brings significant advantages for critically ill patients, for their families, and for the healthcare team

All the benefits linked to the families’ presence into the ICUs greatly outweigh the pandemic risks (Fig. 1), which can be controlled by specific protocols.

## Tips for ICU opening

1. Family members should be allowed in even if only for short periods.

As has been stressed by European experts [6, 25], we must find new ways to keep up active and effective connections between the patient, the family, and the care team. These new strategies must preserve the quantity and quality of the relationship offered to patients and their family, responding to their needs for information and support. Compared to no visit, even short admissions are often of substantial significance to relatives and patients.

Visits to CoViD or non-CoViD ICUs, if properly conducted, do not pose any additional safety risk to patients or visitors [26]; once the visiting protocol has been established, the care team is responsible for its correct application, and for creating conditions "tailored" to the setting, to allow the safest visits.

(2) Different rules should be set for CoViD and non-CoViD ICUs.

In general, simple protocols are needed, shared with the hospital management, to handle family members visiting patients. Relatives should be given clear, well-defined and unambiguous instructions, and their implementation should be carefully supervised.

In CoViD ICUs, family members must be protected with donning-and-doffing protocols, just like healthcare workers. In non-CoViD ICUs, additional health surveillance, appropriate physical protection, and rapid nasopharyngeal swabbing should be considered to protect inpatients from further SARS-CoV-2 illness.

(3) If the total number of family visits must be limited, it is wise to encourage visits for those who can benefit most

There are phases of the disease in which it is more useful, from a clinical point of view, to have a loved one close by, for example during prolonged non-invasive ventilation or during respiratory weaning and the consequent easing of pharmacological sedation [21], and other phases where it is not so important for the patient—though not for the relatives—such as during prolonged deep sedation.

There are moments of hospital stay that are more important from the point of view of his or her *biography*, when the nearness of loved ones is of greater importance, such as the ICU admission, the communication to limit care, or the moments immediately preceding death [27]. Under these inevitably high-stress conditions, physical presence may be more effective in protecting families against psychological trauma.

If it should be necessary to limit the number of admissions for organizational reasons or because of a PPE shortage, it is wise to favor visits that, in the circumstances, can offer the greatest possible benefits to the largest number of both patients and visitors.

(4) It is advisable to set up a special working group in the ICU and to re-evaluate at least monthly the structural and organizational conditions that justify limiting family visits.

The course of the pandemic gives rise to variable levels of hospital overload, with different workloads for operators, different availability of PPE, and different transmission rates at the entire population level. Some decisions to limit visits, which are necessary at certain times, may be inappropriate at others, either by excess or by default. Therefore, it may be useful to prepare different protocols from the outset, with different rules for visits, adapting them automatically, for instance by linking them to national/regional/local regulations.

(5) Relatives and other visitors must be informed about the risks related to accessing CoViD areas.

Personal risk from contact with persons with CoViD can be minimized but will never be zero. All those requesting access to CoViD areas should be aware of the need to balance the possible risks of personal harm with the increased benefits expected from being present beside the patient. Family members and visitors must be fully informed about the risks involved in accessing areas intended for the care of patients with a contagious infectious disease. In this sense, hospital management might be consulted about whether to have visitors entering the CoViD area sign written informed consent.

(6) The re-opening process should be shared with the whole team.

The conditions of "coming out from the pandemic"—hopefully in the next few months—calls for extra attention from an organizational perspective. All staff have experienced work and emotional overload [28] and have inevitably been affected by the changes in internal rules necessitated by isolation. Therefore, it is important that reopening the wards is a procedure shared as far as possible among staff members, since any significant change in the work environment risks increasing stress for healthcare workers [22, 29].

(7) The physical presence of family members should not be limited to ICUs.

The decision to "open" should concern the ICUs together with all the other hospital wards, whose work always precedes or follows that of treating the most critical and complex phase of the disease. This good clinical practice may be harder to manage in settings with fewer doctors and nurses than the ICUs. However, it is advisable to make comprehensive hospital rules to govern the presence of visitors, aiming to restart the process of patient—and family-centered care, even—and especially—during the SARS-CoV-2 pandemic [30].

## How to proceed to opening

The indispensable conditions for visitors to enter the hospital are:

1. The family member and the patient want it.
2. The family member is not in fiduciary home isolation or quarantine, unless ad hoc protocols are in place for these cases, which should in any case be reserved for exceptional times.
3. The family member is asymptomatic and has no risk factors for contagious diseases. Enough PPE is available for the family members.
4. The presence of trained persons (healthcare workers or hospital volunteers) is guaranteed, with the task of indicating internal routes, explaining clearly and in an easily understandable way to non-healthcare professionals how to use the PPE properly, and supervising their correct use.

Given these conditions, we believe the following rules must be applied:

- Use rigorous, agreed procedures to differentiate entry and exit routes, to ensure adequate scheduling, interpersonal distancing, hand washing, and the obligation to wear PPE. When properly implemented, these measures ensure more than adequate safety for patients about the risk of infection due to family visits [31] and protect family members from infection [32].
- Arrange for clinical monitoring: body temperature when the visitor enters the hospital, checking for the absence of flu-like symptoms and other risk factors by completing targeted questionnaires. Optional infectious surveillance with rapid antigenic tests could give an answer within minutes and are not too expensive.
- Schedule visits so that people do not linger in waiting rooms and avoid too many relatives together at the same time. Limit the number of relatives/visitors for each patient and—if it is not feasible to organize a daily visit—consider the possibility of guaranteeing visits by family members at least once or twice a week. This should make it possible to escape from the image of the *"closed"* hospital, even for non-CoViD patients, which nowadays appears frankly unacceptable [14].
- Permit exceptions in circumstances where it is particularly important to allow family members to visit the patient, such as during prolonged hospitalization, in cases of inauspicious short-term prognosis [33], and in all cases of particular patient frailty.

From the organizational point of view, each health facility can organize the pathways to opening as it deems most appropriate and sustainable, modifying internal rules based on continuous monitoring of their effectiveness, with the aim of the earliest possible restoration of good clinical practices of family-centered medicine [13, 14]. Table 1 lists the clinical, cultural, and logistic barriers, with strategies to overcome them.

**Table 1:** The barriers likely to hinder the family presence in the ICU can be overcome by specific strategies

	Barriers to ICU visits	Strategies for implementation
Clinical	Risk of the relatives’ infection when entering CoViD-19 ICUs	Adequate donning and doffing protocols
	Risk of patients’ infection in COVID-free critically ill patients	Screening of family members and visitors before visit
	Inadequate or incomplete PPE employment	Clear instructions and supervision
	Physical discomfort of the visiting family member	Preventive instructions and staff availability
Cultural	Family visits are not a priority for the staff	ICU staff debriefing on family needs and requests
	Lack of motivation and fatigue of staff members	Adequate staff knowledge regarding opening benefits
	Fear of other opportunistic infections brought by families	Literature evidence demonstrating this is not an issue
	Concern of legal consequences in case of visitors’ infection	Informed consent gaining prior to visit
Logistical	Shortage of PPE	If persisting, visits are NOT permitted
	Limited staff availability, time constraints	Extra staff, skilled volunteers
	Lack of protocols/rules	Drafting of hospital/regional shared protocols
	Suboptimal/inadequate space due to surge of ICU admission	Temporary restriction on opening hours and number of visitors

## Generalizability of the present approach

Even if the present paper is based on an Italian setting, the contents are intended to be generalizable. First, the human need for closeness and sharing has been described as scientifically not different among countries [34, 35]. Moreover, the experiences and scenarios used here are from the cultural Italian milieu [4], where the concept of open ICU is much less common than in Northern Europe or North America.

All the contents here presented will be reassessed through a Delphi process as soon as possible, led by the Italian Society of Anesthesia, Analgesia, Reanimation, and Intensive Care Medicine (SIAARTI) and involving a multi-professional taskforce from several Italian scientific societies.

## Conclusions

The SARS-CoV-2 pandemic has unfortunately made it necessary to restrict or prohibit access to hospitals for patients’ relatives. There is ample awareness in the healthcare world of how much suffering these decisions, while necessary, have caused in all those involved: patients and family in the first place, but also doctors and nurses.

Current knowledge and the guaranteed availability of PPE allow us to favor a careful, progressive resumption of opening to family visits, always in full respect of the patient’s wishes. We believe there are no substantial reasons why family members should not be allowed in the CoViD wards: It may be not only useful, but even necessary. In the specific setting of each hospital, all possibilities should be explored to promptly restore good "humanizing" practices in both intensive and non-intensive care units.

## Supplementary Information

**Additional file 1.** Presentation of the social, relational, and organizational challenges due to the pandemic, together with a description of the Authors’ group.

## Data Availability

Not applicable.
