# Supplementary material for: Why and how to open intensive care units to family visits during the pandemic
Source: Crit Care. 2021 Jun 2;25:191. doi: 10.1186/s13054-021-03608-3 (PMC8171999; doi:10.1186/s13054-021-03608-3)
Supplement: Supplementary file 1 — Additional file 1. Presentation of the social, relational, and organizational challenges due to the pandemic, together with a description of the Authors’ group. [file 13054_2021_3608_MOESM1_ESM.pdf]

Why and how to open intensive care units to family visits during the pandemic

Giovanni Mistraletti[1,2], Alberto Giannini[3], Giuseppe Gristina[4], Paolo Malacarne[5], Davide Mazzon[6], Elisabetta Cerutti[7], Alessandro Galazzi[8], Ilaria Giubbilo[9], Marco Vergano[10], Vladimiro Zagrebelsky[11], Luigi Riccioni[12], Giacomo Grasselli[1,13], Silvia Scelsi[14], Maurizio Cecconi[15,16], Flavia Petrini[17].

[1] Department of Pathophysiology and Transplantation, Università degli Studi di Milano, Milan, Italy.
[2] SC Anesthesia and Intensive Care, San Paolo Hospital - Polo Universitario, ASST dei Santi Paolo e Carlo, Milan, Italy.
[3] Unit of Pediatric Anesthesia and Intensive Care, Children's Hospital, ASST Spedali Civili, Brescia, Italy.
[4] Italian Society of Anaesthesia, Analgesia, Reanimation, and Intensive Care Medicine (SIAARTI) Ethics, Rome, Italy.
[5] U.O. Anesthesia and Intensive Care, AOU Pisana, Pisa, Italy.
[6] UOC Anesthesia and Intensive Care, Belluno Hospital, Italy.
[7] Department of Anesthesia and Transplant, Surgical Intensive Care, AOU Ospedali Riuniti, Ancona, Italy.
[8] Direction of Healthcare Professions, Fondazione IRCCS Ca' Granda Ospedale Maggiore Policlinico, Milan, Italy.
[9] General and Neurosurgical ICU, Ospedale dell'Angelo, AULSS 3 Serenissima Veneto, Venice, Italy.
[10] Department of Anesthesia and Intensive Care, San Giovanni Bosco Hospital, Turin, Italy.
[11] Director, Laboratorio dei Diritti Fondamentali, Collegio Carlo Alberto, Turin, Italy.
[12] ICU 4, AO San Camillo-Forlanini, Rome, Italy.
[13] Department of Anesthesia, Critical Care and Emergency, Fondazione IRCCS Ca' Granda Ospedale Maggiore Policlinico, Milan, Italy.
[14] Chair Aniarti, Director of Health Profession Department, IRCCS Istituto Giannina Gaslini, Genoa, Italy.
[15] Department of Biomedical Sciences, Humanitas University, Pieve Emanuele, Italy.
[16] Department of Anesthesia and Intensive Care, IRCCS Humanitas Research Hospital, Rozzano, Italy.
[17] SIAARTI President - Retired Full Professor of Anesthesia and Intensive Care, Chieti-Pescara University, Italy.

ELECTRONIC SUPPLEMENTARY MATERIAL

Social, relational, and organizational challenges due to the pandemic

The pandemic, besides posing a new, serious threat to people's health and life, has also worsened the economic and psychological difficulties [1] of large social groups. In addition, an outsized amount of news, with often conflicting content, has been spread through the mass media about the Corona Virus disease of 2019 (CoViD-19) caused by the new virus. [2] The perception of the work done in hospitals has progressively shifted, and episodes of mistrust and sometimes even hostility towards healthcare professionals are increasingly common. [3]

One must also recall that the freedom to maintain relationships with family members and friends is an expression of the right to private and family life, which is protected by the Italian Constitution and in Europe by the European Convention on Human Rights. It is particularly important in the conditions described here and can be restricted - among other things - to protect health, but always and only within strict limits about what is necessary, and what are the least invasive measures. Moreover, an incomprehensible difference has been created between places open to the public where people can enter, observing certain clear indications, and places of care - even *CoViD-19-free* - where it is absolutely forbidden to enter.

For many health professionals, the impossibility of meeting family members directly, talking to them, enabling them to participate in the care pathways and in decision-making, and above all allowing them to stay close to their loved ones, has contributed to a situation of "moral distress", which is a particular condition in which events force people to act differently from what they consider right. [4]

Re-opening ICUs may lead to work overload for healthcare professionals [5] and can only be achieved in different ways in different settings. However, it can offer some important benefits such as respect for personal rights, sharing of care pathways, greater awareness among family members, less sense of abandon for patients, and less psychological distress for family members and caregivers. [6] It can also lead to less risk of litigation related to remote communication with family members and to the emotional tension resulting from the exclusion of all contact with hospitalized relatives. [7] These benefits are so significant

as to suggest that a path to re-opening for visits should be resumed even if the pandemic indicators worsen again, as happened at the beginning of 2021. In addition, we believe it is appropriate that the hospital as a whole can open up again to family members and people who are significant to patients, encouraging their presence as far as possible beside them. [8]

Authors' group description

The initiative of writing this viewpoint is coming from the Bioethics group of the Italian Society of Anaesthesia, Analgesia, Reanimation, and Intensive Care Medicine (SIAARTI): some of the authors are intensivists physicians, sharing a personal interest for bioethics and participating to the working group on these topics (GM, AGiann, GGrist, DM, MV). Other two intensivist physicians (PM and EC) have been concretely involved in writing protocols for each own hospital, to allow family visits in their CoViD-19 ICUs. The same for two nurses actively promoting projects for ICU opening and humanization (AGalaz and IG).

The intention to write such a paper has been then shared with:

- the chair of the Italian Society of Anaesthesia, Analgesia, Reanimation, and Intensive Care Medicine (SIAARTI): FP,
- the chair of the SIAARTI section of Intensive Care Medicine: GGrass,
- the chair of the SIAARTI Ethics Committee: LR,
- the chair of the European Society of Intensive Care Medicine (ESICM): MC,
- the chair of the Italian Critical Care Nurses Association (ANIARTI): SS,
- a former judge of the European Court of Human Rights: VZ.

All the authors significantly contributed to the development of the contents here presented.

References

1. Ettman CK, Abdalla SM, Cohen GH, Sampson L, Vivier PM, Galea S: **Prevalence of Depression Symptoms in US Adults Before and During the COVID-19 Pandemic**. *JAMA network open* 2020, **3**(9):e2019686.
2. Azoulay E, Curtis JR, Kentish-Barnes N: **Ten reasons for focusing on the care we provide for family members of critically ill patients with COVID-19**. *Intensive care medicine* 2020.
3. Alonso-Ovies A, Heras La Calle G: **ICU: a branch of hell?** *Intensive care medicine* 2016, **42**(4):591-592.
4. Azoulay E, De Waele J, Ferrer R, Staudinger T, Borkowska M, Povoa P, Iliopoulou K, Artigas A, Schaller SJ, Hari MS *et al*: **Symptoms of burnout in intensive care unit specialists facing the COVID-19 outbreak**. *Annals of intensive care* 2020, **10**(1):110.
5. Giannini A, Miccinesi G, Prandi E, Buzzoni C, Borreani C, Group OS: **Partial liberalization of visiting policies and ICU staff: a before-and-after study**. *Intensive care medicine* 2013, **39**(12):2180-2187.
6. Raudenska J, Steinerova V, Javurkova A, Urits I, Kaye AD, Viswanath O, Varrassi G: **Occupational burnout syndrome and post-traumatic stress among healthcare professionals during the novel coronavirus disease 2019 (COVID-19) pandemic**. *Best practice & research Clinical anaesthesiology* 2020, **34**(3):553-560.
7. Hart JL, Turnbull AE, Oppenheim IM, Courtright KR: **Family-Centered Care During the COVID-19 Era**. *Journal of pain and symptom management* 2020, **60**(2):e93-e97.
8. Munshi L, Evans G, Razak F: **The case for relaxing no-visitor policies in hospitals during the ongoing COVID-19 pandemic**. *CMAJ : Canadian Medical Association journal = journal de l'Association medicale canadienne* 2020.
